# Epigenetics and Gut Microbiota Crosstalk: A potential Factor in Pathogenesis of Cardiovascular Disorders

**DOI:** 10.3390/bioengineering9120798

**Published:** 2022-12-13

**Authors:** Vineet Mehta, Priyanka Nagu, Baskaran Stephen Inbaraj, Minaxi Sharma, Arun Parashar, Kandi Sridhar

**Affiliations:** 1 Department of Pharmacology, Govt. College of Pharmacy, Rohru 171207, Himachal Pradesh, India; 2 Department of Pharmaceutics, Govt. College of Pharmacy, Rohru 171207, Himachal Pradesh, India; 3 Department of Pharmacy, Shri Jagdishprasad Jhabarmal Tibrewala University, Jhunjhunu 333001, Rajasthan, India; 4 Department of Food Science, Fu Jen Catholic University, New Taipei City 242 05, Taiwan; 5 Haute Ecole Provinciale de Hainaut-Condorcet, 11, Rue de la Sucrerie, 7800 Ath, Belgium; 6 Faculty of Pharmaceutical Sciences, Shoolini University of Biotechnology and Management Sciences, Solan 173229, Himachal Pradesh, India; 7 Department of Food Technology, Koneru Lakshmaiah Education Foundation Deemed to Be University, Vaddeswaram 522502, Andhra Pradesh, India

**Keywords:** epigenetics, gut microbiota, cardiovascular disorders, DNA methylation, histone modification, miRNA

## Abstract

Cardiovascular diseases (CVD) are the leading cause of mortality, morbidity, and “sudden death” globally. Environmental and lifestyle factors play important roles in CVD susceptibility, but the link between environmental factors and genetics is not fully established. Epigenetic influence during CVDs is becoming more evident as its direct involvement has been reported. The discovery of epigenetic mechanisms, such as DNA methylation and histone modification, suggested that external factors could alter gene expression to modulate human health. These external factors also influence our gut microbiota (GM), which participates in multiple metabolic processes in our body. Evidence suggests a high association of GM with CVDs. Although the exact mechanism remains unclear, the influence of GM over the epigenetic mechanisms could be one potential pathway in CVD etiology. Both epigenetics and GM are dynamic processes and vary with age and environment. Changes in the composition of GM have been found to underlie the pathogenesis of metabolic diseases via modulating epigenetic changes in the form of DNA methylation, histone modifications, and regulation of non-coding RNAs. Several metabolites produced by the GM, including short-chain fatty acids, folates, biotin, and trimethylamine-N-oxide, have the potential to regulate epigenetics, apart from playing a vital role in normal physiological processes. The role of GM and epigenetics in CVDs are promising areas of research, and important insights in the field of early diagnosis and therapeutic approaches might appear soon.

## 1. Introduction

Cardiovascular diseases (CVDs) are the leading causes of mortality globally. CVDs are a diverse array of disorders associated with the heart and blood vessels. Some of the common ones are coronary heart disease, hypertension, cerebrovascular disease, atherosclerosis, myocardial infarction, ischemia/reperfusion injury, stroke, and heart failure, among others. Myocardial infarction and stroke accounted for 85% of death in CVDs [1]. CVDs are not just an enormous health concern but a significant financial threat, with trillions of dollars’ worth of economic strain.

The mechanisms underlying the pathophysiology of CVDs are complex and far from complete understanding. Epigenetics has recently been identified as a potential area of research in CVDs and has helped decipher the role of environmental influence over genetics in pathological conditions [2]. “Epigenetics” (“epi”, meaning “upon”, “on”, or “around” and the word “genetics”) can be defined as the mechanisms that affect gene expression and function without altering the genome. It is a crucial process regulating chromatin architecture, gene expression, and defining the cell’s phenotype, and thus, it is directly involved in normal development and diseased state. Epigenetic markers are hallmarks of various disorders and diseased states, such as diabetes mellitus, autoimmune diseases, infectious diseases, cancer, CVDs, and several other disorders, which makes them potential targets for new drug therapies. There are three main epigenetic processes viz DNA methylation, chromatin remodelling (posttranslational histone modifications), and RNA-based mechanisms such as microRNAs (miRNAs) and long non-coding RNAs (LncRNAs) [3]. All these mechanisms work harmoniously to regulate the body’s normal physiological functions. Epigenetic causality of CVDs is a relatively new and promising field that has provided diverse yet critical insights. Occasionally, environmental factors have a key role in CVD pathogenesis. Epigenetics addresses, to a certain extent, the underlying processes for the effect of the environment on genetics and, consequently, how these effects may be handed down down the generations [4]. Epigenetics also answers the variability in susceptibility to CVDs among different individuals, forming the basis of biomarkers and new therapeutic strategy development.

With new technologies such as methylation arrays, studying epigenome-wide variation has become cheaper and less tedious. Although a relatively nascent field, epigenetics has already unravelled new mechanisms underlying the development of CVDs, such as atherosclerosis, thrombosis, and inflammation. Further, the success of epigenetics in oncology has provided us with promising proof of concept [5,6].

Besides influencing epigenetics, environmental factors such as diet are also known to significantly alter the physiology of the microbiota present in the human gut. Human gut microbiota (GM) is another crucial factor significantly associated with CVDs. Our gut harbors more than 100 trillion microbes, ten times the number of human cells. Past decades have unfolded the immense role of GM in human health and several diseased states. Although most of the data regarding GM dysbiosis in CVDs is associative, we believe it could very well be the underlying cause. GM dysbiosis has been associated with several CVD pathologies, such as atherosclerosis, heart failure, etc., and predisposing factors such as obesity and hypertension. GM-derived metabolites such as trimethylamine (TMA)/trimethylamine-*N*-oxide (TMAO), short-chain fatty acids (SCFAs), and primary and secondary bile acids are suggested to potentially contribute to the pathogenesis of CVDs [7].

Considering the close association of GM and epigenetics with environmental factors [8], the interplay of GM and epigenetic factors in disease progression cannot be neglected. GM-induced epigenetic modifications could be a potential mechanism affecting human health and diseases. GM modulates several non-genetic factors, such as body weight, metabolism, physical activity, dietary factors, environmental toxins, etc., which directly affect epigenetic modulation of gene expression [9]. GM-produced metabolites such as SCFAs, folates, biotin, and TMAO act as critical co-factors and allosteric regulators of enzymes such as methylases and acetylases, which ultimately modulate epigenetic processes [7]. Further, the symbiotic association of microbes and humans has co-evolved for over a million years, and considering the complexity of the human genome and gut microbiome (genome of gut microbiota), it is plausible that epigenetics could connect these two genomes via complex mechanisms [9]. Accumulated evidence from the recent past suggests that both GM and epigenetics hold enormous potential to modulate human health, and their interplay has started to emerge [10,11,12]. This connection between GM and epigenetics could help in a better understanding of the pathogenesis of CVD. Therefore, this narrative review aims at assessing gut microbiota’s possible role in CVD aetiology via epigenetic mechanisms.

## 2. Epigenetic Mechanisms in CVDs

### 2.1. DNA Methylation

DNA methylation is the most explored epigenetic mechanism in the mammalian genome. DNA methylation refers to the addition of a methyl group to the fifth carbon of cytosine. The major site of DNA methylation lies in the CpG (cytosine-phosphate-guanine) islands found in the 5′ regulatory regions of genes. The enzymes involved in DNA methylation are called DNA methyltransferases (DNMTs) [13]. A nonprotein-forming amino acid, homocysteine, is critical in the methylation cycle and likely an underlying factor in CVD pathogenesis [14]. High levels of S-adenosyl homocysteine, which inhibit transmethylation reactions and reduce methylation throughout the epigenome, are associated with increased intake of homocysteine [15]. Hypomethylation has been associated with conditions such as autoimmune dysregulation and poor prognosis in cancer patients [16,17,18], while the association with CVDs is unclear. Long interspersed nuclear element (LINE-1) methylation studies have found inverse associations with the risk of CVDs [19,20,21,22], which might possess biomarker potential for early diagnosis [21]. In contrast, Alu methylation or HpaII/MSPI restriction enzyme ratio, often used to quantify global methylation, was found to be positively associated with adverse cardiovascular outcomes [23,24,25,26].

The global methylation studies have a drawback of low resolution, which is why they give a vague idea about epigenetic impact on CVDs. Individual gene-specific methylation studies are more precise and provide solid evidence supporting DNA methylation in the development of cardiovascular disease [9]. Reduced methylation of inflammatory pathway genes has been reported to be associated with enhanced gene expression and decreased circulating levels of pro-inflammatory cytokines, a potential biomarker of cardiovascular dysfunction [9]. In leukocytes from acute coronary syndrome patients compared to healthy controls, a methylation study of a pro-inflammatory protein-encoding gene ANGPTL2 revealed a decreased promoter methylation associated with higher ANGPTL2 [27]. Another study reported that reduced IL-6 gene methylation was associated with higher IL-6 plasma levels in ischemic heart disease patients [28]. Hypermethylation-induced decreased expression of the inflammatory pathway-associated ASC gene was associated with improved outcomes in patients with heart failure [29]. Other than the inflammatory pathways, DNA methylation is associated with CVD pathologies such as hypertension, dyslipidemia, and obesity. Changes in methylation of troponin encoding gene TNNT1 are associated with increased high-density lipoprotein cholesterol (HDL-C) and a significant risk of coronary artery disease [30]. Similarly, the methylation of the IGF2 (Insulin-Like Growth Factor 2) gene is associated with a higher ratio of Triglycerides to HDL-C and obesity [31,32]. In umbilical cord blood, hypermethylation of POMC, a gene encoding proopiomelanocortin, was associated with higher blood triglycerides and insulin during childhood [33]. The methylation of genes encoding pro-inflammatory proteins such as TLR2, iNOS, and IFN-γ was associated with fluctuations in blood pressure in the elderly [34]. Hypermethylation of the HSD11B2 gene promoter led to increased activity of the 11-betaHSD2 enzyme and the risk of hypertension [34]. The epigenetic effects of the FTO gene, a key genetic predictor of obesity and CVD risk factor, stand out among other potential genes [35]. The FTO protein functions as an N6-methyladenosine demethylase, accelerating RNA changes required for preadipocyte development [36,37] and hence increasing obesity, a significant CVD risk factor. FTO also exerts long-term effects on the promoter of the homeobox gene IRX3, reducing thermogenesis, and promoting lipid storage [38].

Interestingly, while the deleterious effect of FTO sequence variation on obesity was buffered by physical activity [39], a six-month exercise intervention failed to change FTO methylation levels in adipocytes [40], suggesting alternative mechanisms.

The methylation of another gene, F2RL3, a tobacco-related methylation site, was perhaps connected with inflammation and obesity [41]. Hypermethylation in INS and GNASAS, thought to be programmed by the intrauterine environment, predicted myocardial infarction (MI) risk in women but not in males [42]. In addition, higher methylation of PLA2G7 was related to coronary heart disease in women in relation to age and lipoprotein characteristics [43]. Higher methylation of a locus in the CVD-linked 9p21 region was seen in coronary artery disease [44], suggesting an epigenetic basis for DNA sequence variation.

With the help of advanced methylation array technology, studies have found epigenome-wide markers of CVDs, including coronary artery disease [45,46], ischemic stroke [47], cardiomyopathy [48,49], and cardiovascular mortality [50]. Four out of more than 16,000 CpG sites, namely STRADA (STE20-related kinase adaptor alpha), C1QL4 (Complement C1q Like 4), HSP90B3P (heat shock protein 90 kDa beta), and KCNQ1 (Potassium Voltage-Gated Channel Subfamily Q Member 1) have shown associations with CVD phenotypes. Subsequent studies have highlighted the role of KCNQ1 in coronary endothelial dysfunction [51] and atrial fibrillation [52]. In Zebra fish model, it was observed that methylation of ADORA2A (adenosine A2a receptor) and LY75 (lymphocyte antigens) were associated with human dilated cardiomyopathy [49].

Epigenetic studies have been fruitful in identifying numerous biologically relevant targets, and to some extent, addressed the effect of environmental factors, such as metabolism, smoking, air pollution, diet, etc., on CVDs. Multiple studies have found robust relationships between methylation and expression of CPT1A, a gene encoding a key enzyme in the fatty acid metabolism pathway, and body mass index (BMI), lipoprotein profiles, and metabolic syndrome [53,54,55,56,57]. A similar *in-utero* study revealed that differential methylation at that locus correlated with birth weight and plasma lipids, suggesting prenatal nutrition lays the path for epigenetic programming of metabolic pathways [58]. Methylation of ABCG1, a gene encoding a protein essential to TG metabolism, showed association with critical cardiometabolic parameters, including hypertriglyceridemic waist [55], obesity [56], glucose and insulin profiles [59,60], type 2 diabetes [61], and plasma lipid profiles [62].

In smokers, epigenome-wide studies have found methylation locus in the AHRR gene (aryl hydrocarbon receptor repressor) of monocytes to be significantly associated with subclinical atherosclerosis [63]. Methylation of THBS1 (thrombospondin 1), another smoking-related locus, was found to correlate with plasma thrombospondin-1 levels [64], although the impact of thrombospondins on CVD risk remains contentious [65]. Air pollution is also strongly associated with CVDs [66]. An epigenome-wide methylation study observed the involvement of several genes in oxidative stress and inflammatory pathways, on exposure to fine particulate matter [67].

Dietary effect on cardiovascular effects also sees DNA methylation as a critical mechanism. According to an in-vitro investigation, oleic and arachidonic acid had unique effects on monocyte epigenomes, with the latter being enriched for atherosclerosis-related variations [68]. The dietary presence of methyl-rich nutrients such as methionine, one-carbon units, and choline might leave an epigenetic impact on CVD outcomes, since DNA methylation depends on it [69]. An animal study showed that a methionine-rich diet had pro-atherogenic effect via the fatty acid binding protein demethylation pathway [70]. How different dietary methyl donors shape the epigenetic response is still unclear and requires future research. Therefore, the impact of environmental factors on the pathogenesis of CVDs via epigenetics pathways and GM cannot be overlooked.

Although cost-effective and robust in deciphering the methylome, epigenetic studies require cautious interpretation and validation to eliminate false positive results. The epigenome-wide methylation studies have given incriminating evidence that the well-known metabolic, lifestyle, and environmental factors underlie the pathology of CVDs via epigenetic modulation.

### 2.2. Chromatin Remodelling and Histone Modifications

Chromatin remodeling refers to a change in chromatin to a transcriptionally accessible state from a condensed form, allowing transcription factors and DNA-binding proteins to access and control gene expression. Epigenetic modifications of histone proteins via three sets of enzymes viz histone methyltransferases (HMTs), histone deacetylases (HDACs), and histone acetyltransferase (HATs) and, can alter chromatin-dependent processes such as gene expression, DNA replication, and repair [71]. HATs, HDACs, and HMTs contribute to a variety of cardiovascular abnormalities by activating or repressing gene transcription. According to the severity of the plaque in carotid arteries, histone methylation (transcriptional repressor) and acetylation (transcriptional activator) exhibit unique patterns [72]. Cell culture and animal models have demonstrated that blocking HDACs promotes hyperacetylation of histones, resulting in decreased inflammation and atherogenesis [73], mediated through the expression of ABCA1 and ABCG1 genes, the latter of which is a significant epigenetic predictor of plasma cholesterol [62]. Further, histone modifications also affect gene expression of pathways involved in inflammation [74,75], hypertension [76,77], diabetic cardiomyopathy [78], cardiac hypertrophy [79], ischemic myocardial repair [80], stroke [81], heart failure [82], and abdominal aortic aneurysm [83]. Further, cellular fate in cardiac morphogenesis is affected by interactions between transcription factors and histone modifiers [84]. Their relative ease of inhibition and diverse effects on cardiovascular physiology make histone modifiers prospective diagnostic/prognostic/therapeutic targets for CVDs.

### 2.3. RNA-Based Mechanisms

With 98% of our genome being non-coding, previously considered to be non-functional, it is now realized that it is actively transcribed to produce thousands of non-coding transcripts, including microRNAs (miRNAs) and long non-coding RNAs (LncRNAs). miRNAs are a class of small non-coding RNAs (~22 nucleotides) that were first discovered in *Caenorhabditis elegans* [85,86]. More than 2000 miRNAs have been found in humans, many of which are evolutionarily conserved; miRNAs profoundly affect gene expression as they pair with a specific complementary sequence of mRNAs, resulting in degradation and inhibition of translation. In fact, miRNAs have demonstrated a wide range of biological activities and are now implied in almost every diseased state [87,88]. LncRNAs are classified as all ncRNAs longer than 200 nucleotides. LncRNAs are very heterogeneous and exhibit diverse biological functions via interacting with various other RNAs or proteins. LncRNAs can interfere with transcriptional and post-transcriptional gene regulation, as well as mRNA translation, depending on their subcellular localization in the nucleus or cytoplasm, respectively. Recent years have shown that miRNAs and LncRNAs have immense biomarker and therapeutic potential in diseased states, including CVD etiology [87,88]; miRNAs are highly stable in plasma [89] and can be exploited for biomarker and therapeutic targeting. Although the precise miRNA fingerprint of CVDs is still unclear, studies have found that miR-1 and 133 levels are higher in angina pectoris, MI, and acute coronary syndromes [90]. Both miR-1 and miR-133 are cardiac myocyte-specific miRNAs involved in the development of the heart and are observed to be dysregulated in heart failure [91]. Indeed, miR-1 is involved in regulating the expression of heat shock protein 90 aa1 (HSP90AA1), which attenuates oxygen-glucose deprivation-induced apoptosis of ventricular cells in ischemia/reperfusion injury [92], and modulates arrhythmias via several ion channels and conduction-related proteins [93]. Similarly, miR-133 is implicated in cardiac arrhythmias, hypertrophy, and apoptosis [94]. Another miRNA, miR-499, was more sensitive than cardiac troponin T as a biomarker for acute non-ST segment elevation MI [95]; miR-499 exerted cardioprotective effects by regulating calcineurin and, thus, cardiac remodeling in the setting of ischemia [96]. Some other highly pleiotropic miRNAs involved in acute heart disease include miR-208 [97], miR-23 [98], miR-214 [99], miR-21 [100], miR-126, miR-197, miR-223 [101], and possibly many more. The miRNA-based mechanisms are also critically involved in intermediate-risk phenotypes; for example, miR-148 is involved in lipid metabolism by varying HDL, LDL, and cholesterol levels [102,103]; miRNAs also regulate endothelial function, plaque development and rupture, and blood vessel formation, thus governing the progression of atherosclerosis [104]. Indeed, miRNAs are also associated with hypertension; for example, binding of miR-425 decreased atrial natriuretic factor (encoded by Nppa gene) expression and lowered the circulating levels of the corresponding protein; interestingly, such binding does not occur in the presence of the G allele at the rs5068 locus of Nppa gene, previously linked to blood pressure [105]. In fact, miRNAs are now the most promising biomarkers for CVDs because of their high specificity and sensitivity. They also seem to have strong prognostic values, enhancing risk stratification, and accurate prediction of the cardiovascular risk patients.

## 3. Gut Microbiota and CVD: A Role of Heart-Gut Axis

The human gut microbiota, with more than 100 trillion microbial cells, is the community of microorganisms living in the small and, mainly, the large intestine of humans. Often referred to as our forgotten organ, this microbial ecosystem has co-evolved with humans for millions of years and plays an integral role in modulating human physiology. GM benefits the host via its diverse array of functions, including the synthesis of essential vitamins (vitamins K, B12, folate, and thiamine), energy harvesting from undigested fiber via fermentation, production of the host-absorbable SCFAs, propionate, acetate, and butyrate, and metabolic processing (mainly hydrolysis and reduction) of various non-dietary xenobiotic compounds entering the gut [106,107]. Ongoing research suggests that intestinal bacteria have a crucial role in both the maintenance of health and the onset of disease. Heart-gut axis is a new promising filed unraveling the role of gut microbiota in CVD etiology. Mounting evidence over the past decade has established a significant association of inter- and intra-individual variation and versatility of the GM composition with health and various disease states, including CVDs. Although GM dysbiosis is clearly observed in patients with CVDs, and thus most of the data is mainly associative, few studies have supported a causal link between GM and CVDs. GM transplantation studies have shown that specific pathways and metabolites influence host metabolism and CVDs, sometimes via specific host receptors [108]. Significant advancements in sequencing and bioinformatics have led to the association of dysbiosis with several diseased states. Initially, GM-related studies observed that fecal microbial community composition was associated with the development of obesity and insulin resistance [109,110,111]. Subsequently, it was discovered that GM dysbiosis at an early age increased the risk of adiposity [112]. GM compositional changes have been reported in several CVD phenotypes, including hypertension, dyslipidemia, insulin resistance, and other metabolic phenotypes [113,114]. Further, it was observed that human atherosclerotic plaques contained bacterial DNA, although the origin of bacteria in the walls of the artery was not confirmed to be of the gut but was suspected [115]. The most intriguing causal link between GM and CVD came from a study where it was observed that the production of trimethylamine N-oxide (TMAO), a GM-produced metabolite, following ingestion of western dietary nutrients (e.g., lecithin, choline, carnitine) [116,117,118]. TMAO is found to be a biomarker for CVD risk and accelerated in multiple clinical cohorts and animal models [118]. Using germ-free (GF) mice, a recent fecal microbial transplantation study found a direct association of elevated circulating TMA and TMAO levels with an enhanced rate of thrombus formation and reduced time to cessation of blood flow following arterial injury in vivo [119].

### 3.1. RNA-Based Mechanisms

It was Ott SJ et al. in 2006 who identified the presence of microbial DNA in atherosclerotic plaques and concluded that bacterial colonization could accelerate disease progression [120]. Korean et al. found that the microbial flora of atherosclerotic plaque had specific microbial species which were highly diverse and variable between individuals. They also found that *Chryseomonas* levels in the gut correlated with plasma cholesterol levels, which might contribute to atherosclerosis development and/or progression [121]. Jie et al. observed that *Enterobacteriaceae* and *Streptococcus* spp. were higher in atherosclerotic CVD [122]. Karlsson et al. observed an increase in microbes of the genus *Collinsella* and a decrease in *Eubacterium* and *Roseburia* in patients with symptomatic atherosclerosis [123]. Ziganshin et al. found that members of the *Burkholderiales* were at high levels in all atherosclerotic plaques [124]. Similarly, Davies et al. observed that *Pseudomonas* spp. biofilm was present in atherosclerotic plaques [125]. Animal studies have too confirmed the involvement of microbes in atherosclerosis. *Lactobacillus rhamnosus* GG (LGG) was found to have protective effects on atherosclerotic plaque size [126]. They also observed that five bacteria genera namely *Eubacerium, Anaeroplasma, Oscillospira, Roseburia,* and *Dehalobacterium* showed a protective against atherosclerosis [126]. Another study showed that even when fed with a low-cholesterol diet, GF mice showed accelerated development of atherosclerosis [127], indicating the protective effect of GM. Similarly, a study showed that the absence of microbiota increased plasma cholesterol levels and atherosclerotic lesions [128]. Several other studies have also linked some bacteria, such as *Porphyromonas gingivalis* and *Aggregatibacter actinomycetemcomitans*, to the accelerated development of atherosclerosis in dietary intervention-based animal models [129,130,131].

These findings suggest that GM somehow contributes to the progression of atherosclerosis, with some microbes being causative while some are preventive. The underlying mechanism by which microbes initiate or impede the development of atherosclerosis is unclear and requires detailed investigation. One possible mechanism could be the ability of GM to produce bile acids and hence modulate lipid metabolism.

### 3.2. Intestinal Barrier Dysfunction in CVDs

Gut leakiness is another potential gateway for the translocation of GM-derived products into host circulation, causing inflammation. In its healthy state, intestinal barrier function is maintained by physical factors, including tight junctions between epithelial cells, mucus production, and mucosal immunity. Bowel wall edema and impaired barrier function are often observed in heart failure (HF) patients [132,133]. This leaky gut concept has been validated in multiple studies of HF patients, wherein they are observed to have altered intestinal integrity and subsequent elevated systemic levels of proinflammatory cytokines, which correlates with clinical severity and poor prognosis [134,135]. Further, when the gut barrier is impaired, lipopolysaccharide (LPS) from gram-negative bacteria can enter the host circulation and orchestrate a proinflammatory state in the host [136]. Elevated LPS and other bacterial products have been mechanistically linked to the modulation of inflammation, immunity, and vascular function [108]. Decompensated HF patients were observed to have higher blood endotoxin levels compared with stable counterparts [137]. Furthermore, a study observed a biomarker role of LPS for major adverse cardiac events in a cohort of patients with atrial fibrillation, suggesting GM-associated endotoxins impact CVD complications [138]. It is important to understand that various factors beyond gut leakiness contribute to CVD. There is a complex relationship between GM dysbiosis, intestinal integrity, host systemic inflammation, and CVD susceptibility. It is noteworthy that although CVDs have been linked to endotoxemia, gut barrier defects caused by colitis and inflammatory bowel diseases are not generally associated with CVD risks. Further research is required to understand the role of gut bacterial proinflammatory factors in triggering systemic inflammatory cascades that can be exploited as therapeutic tools for risk prediction and better management of CVDs.

### 3.3. GM-Produced Metabolites in CVDs

#### 3.3.1. Bile Acids

Another influencer over human physiology are bile acids (BAs) which are predominantly GM-derived and modulate host metabolism. BAs, mostly known for emulsification and adsorption of lipids, are composed of a diverse array of structurally specific species with varying inter-individual concentrations. Recent studies show that besides lipid metabolism, BAs play a role in glucose/insulin metabolism and inflammation. A small fraction of the total BA pool is the one synthesized in the liver, which, once secreted in the duodenum, is modified by GM into a remarkably large array of BA species. BAs have the potential to modulate GM composition via their potent antimicrobial and immune response properties [139]. Similarly, bile obstruction can lead to bacterial overgrowth syndromes [140]. Interactions among diet, GM, and specific BAs are complex and dynamic, and any disturbances could contribute to CVD phenotypes and disease susceptibly. Studies have revealed that plasma BA levels were associated with insulin resistance in type 2 diabetes mellitus [141,142], and modulation of BA levels could be exploited as an adjuvant strategy for diabetic treatments [143]. Although this strategy seems less appealing, a mechanistic association between BA levels and CVD pathogenesis could still be of some use.

#### 3.3.2. Short-Chain Fatty Acids (SCFAs)

SCFAs are the most studied GM-derived metabolites that have profound physiological effects. SCFAs are fatty acids with five or fewer carbon atoms and are produced in large quantities by GM through anaerobic fermentation of dietary fiber [144,145]. The most common SCFAs include acetate, propionate, and butyrate, which have been involved in the homeostasis of critical cardiovascular parameters such as blood pressure, myocardial repair, and inflammation. These SCFAs are produced mainly by some bacteria of genera’s, including anaerobic *Bacteroides*, *Bifidobacterium*, *Eubacterium*, *Streptococcus*, and *Lactobacillus* [146,147]. SCFAs are also produced by host cell metabolism (e.g., acetate) [148], but the fact that the SCFA levels in GF-mice are undetectable speaks for itself [149]. SCFAs exert a direct and indirect role in a diverse array of functions such as energy source (for the colonocytes of the intestine), lipid metabolism [112], glucose homeostasis, gut inflammation, and neurogenesis [150]. An influx of SCFAs to the liver resulted in significant changes in the regulation of hepatic lipids in the obese phenotype. A study by Cho et al. showed that SCFAs are associated with adiposity in early life [112]. Another study reported that antibiotics exposure during weaning led to dysbiosis with the increasing metabolic capacity to produce acetate, propionate, and butyrate [151]. Antibiotics-induced dysbiosis in early life [152] is also linked with reduced host immunity [153] and cardiometabolic diseases, such as diabetes mellitus [154,155]. Preliminary clinical studies have observed that fiber intake is associated with a decrease in blood pressure [156], which could be because GM ferments it to produce SCFAs. Studies observed a differential action of propionate on blood pressure via different mechanisms of administration [157]. By depleting GM composition post-antibiotic exposure, the resultant SCFA levels dropped and a subsequent increase in blood pressure was observed, corroborating the homeostatic role of SCFA in blood pressure regulation [157,158,159]. However, the overall hypotensive effects of SCFAs could be due to their ability to reduce cardiac output and vascular resistance [160]. Another study observed that fecal transplantation from human hypertensive patients caused hypertension in GF-mice [161]. In a contradicting study, fecal transplantation from normotensive rats aggravated hypertension in hypertensive rats, suggesting that additional host variables may interact with microbial factors to modulate blood pressure [162]. SCFAs have also been implicated in hypertensive end-organ damage in angiotensin II–infused mice [163]. These and numerous other studies indicate that SCFAs are critical in modulating vasomotor tone and blood pressure. Further, studies have indicated that SCFAs are involved in other CVD processes, such as ischemia-reperfusion injury, cardiac repair following myocardial infraction, and impaired arterial compliance [164,165].

#### 3.3.3. Trimethylamine N-Oxide (TMAO)

It was one of the first studies by Wang et al. that casually linked GM-derived TMAO production post-ingestion of specific dietary nutrients (e.g., lecithin, choline, carnitine) to CVD risk [116,117,118]. TMAO was found to have both predictive capability and a positive correlation to atherosclerosis development [116,117,118], which casually linked GM-derived TMAO production post-ingestion of specific dietary nutrients (e.g., lecithin, choline, carnitine) to CVD risk. Dietary precursors for TMAO production include choline [116,117,118], phosphatidylcholine [117], and carnitine [116]. These nutrients are a common part of the western diet, including meat products. Various TMA and TMAO precursors such as betaine, γ-butyrobetaine [166], and trimethyl lysine [167], have shown good prognostic association with CVD risk [118]. TMAO has been observed to augment atherosclerosis in various preclinical studies [168,169,170,171,172]. It has also been linked to increased thrombosis by promoting platelet reactivity [173,174,175], vascular inflammation and inflammasome activation [176,177,178], increased risk of HF [179,180,181], and chronic kidney disease [182,183,184]. Systemic TMAO levels have been exploited as a biomarker for predicting outcomes in several CVD phenotypes, such as peripheral artery disease [185], coronary artery disease [186], acute coronary syndrome [186,187], and heart failure [188,189,190]. These data suggest the prognostic potential of TMAO and a high degree of association with several forms of CVDs.

#### 3.3.4. Phenylacetylglutamine (PAG)

PAG, a phenylalanine-derived metabolite, is a recently identified GM-produced metabolite associated with CVD risk. Since type II diabetes mellitus (T2DM) is a well-known but poorly stratified risk factor for CVDs, Nemet et al. performed a study to identify novel metabolites showing a correlation with CVDs in T2DM subjects and found PAG as one of the potential candidates. The study observed that PAG was associated with major adverse cardiac events such as heart attack, stroke, and death in both diabetics and non-diabetics [191]. Transplanting genetically engineered microbes in the intestines of GF mice has further confirmed the role of PAG in host platelet reactivity and in vivo thrombosis potential [192,193]. It is fascinating how GM directly or indirectly modulates several CVD phenotypes. The fact that the absence of GM amends homeostasis of critical cardiovascular events, such as blood pressure [194], post-infarction myocardial repair [164], and thrombosis growth [195], etc., speaks for itself. Although further investigations are required to solidify the PAG-mediated CVD pathologies, this could potentially be another GM-based pharmacological target [196].

## 4. Gut Microbiota and Epigenetics: Potential Interaction during CVDs

**Figure 1:**
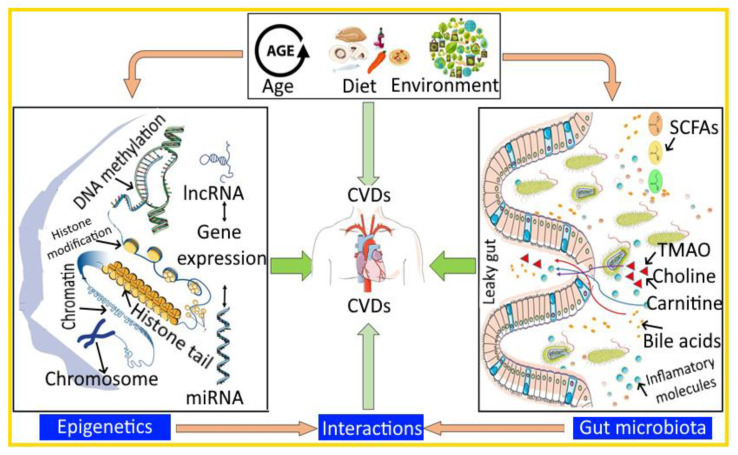
Diagrammatic representation of the interaction between gut microbiota (GM) and epigenetics during CVDs. DNA, deoxyribonucleic acid; lncRNA, long non-coding RNAs; miRNA, microRNA; CVDs, cardiovascular diseases; SCFAs, short-chain fatty acids; TMAO, trimethylamine-*N*-oxide.

The human gut microbiome has evolved along with humans for millions of years and has established a very diverse and complex symbiotic association, most of which is not understood. The interaction between human GIT and infective agents alters the physiological and pathogenetic processes via different molecular mechanisms. One such mechanism is the epigenetic regulation of host physiological processes by modulated metabolic activities of the GM. Interestingly, both GM and epigenetics are dynamic processes and are regulated to a great extent by the environment and diet, which suggests that both of them could have common triggers and could work in association to regulate host physiology [197,198,199]. The interaction of the GM-epigenetics during the CVDs is depicted in Figure 1. Moreover, the primary enzymes involved in the epigenetic regulations are acetylases and methylases, and the activity of these enzymes is dependent on the various metabolites generated from the gut of the host that act as co-factors and substrates for these enzymes [197,198,199]. GM can affect the epigenetic processes of the body at different stages of life, and thus could critically regulate health and disease [200]. Diverse anatomical sites have been explored to evaluate the relationship between microbiome and epigenetics, and their role in conditions such as autoimmune disease, cardiovascular disorders, and cancer, etc. [200]. The pattern and development of different types of diseases can be well understood by investigating the epigenetic relationship of microbiota [201]. Lifestyle, microflora exposure, etc. have been considered to be significant causes for the occurrence of health disorders, such as obesity, allergies, asthma, cancer, etc., as per various epidemiological studies [200]. Furthermore, aging, obesity, dietary changes, and administration of food products with high amounts of calories, can significantly affect the epigenetic makeup, and enhance the risk to develop various diseases [9,202]. Interestingly, exposure to microbiome in the fetal stage can influence the developmental processes, and also, exposure to the maternal microbiome can develop disease risks in later stages of life [203,204].

Age, dietary, and environmental factors not only play a crucial role in the pathogenesis of CVDs, but are know to significantly modulate epigenetics and GM profile of an individual. Epigenetics is known to be associated with CVDs via mechanisms such as DNA methylation, histone modification, miRNA, LncRNAs, etc. On the other hand, GM modulates the pathogenesis of CVDs via metabolites such as SCFAs, TMAO, bile acids, inflammatory mediators, etc. Interestingly, both epigenetics and GM are dynamic processes, but their interaction and bidirectional relationship to modulate the pathogenesis of CVDs has now started to emerge.

Diagrammatic representation of the interaction between gut microbiota (GM) and epigenetics during CVDs. DNA, deoxyribonucleic acid; lncRNA, long non-coding RNAs; miRNA, microRNA; CVDs, cardiovascular diseases; SCFAs, short-chain fatty acids; TMAO, trimethylamine-*N*-oxide.

Studies investigating the interplay of GM and epigenetics regulating CVDs are lacking. However, the mechanistic insights into these phenomena clearly suggest that the interplay could play a crucial role in the development and progression of CVDs. The growing body of evidence further suggests the direct association of the metabolites generated from the human gut with the various epigenetic processes [205]. Epigenetic mechanisms are under the influence of environmental factors and diet, and thus any changes in these factors could lead to the alteration in the normal body’s physiological gene expression and disease state [206]. These epigenetic factors are most dynamic during early childhood when the GM of the baby is being developed, and therefore epigenetics could be related to the development and colonization of the human gut during early childhood through a variety of processes that include diet, breastfeeding, antibiotic treatments, infections, etc.

Further, metabolites from the gut, such as SCFAs are known to inhibit the activity of the HDAC and therefore modulate the associated gene expression [207]. Likewise, several microbiota-dependent changes in the chromatin that were observed after polysaccharide-rich diet consumption were prevented by the consumption of the Western-style diet [208]. Further, administration of the SCFAs to the GF-mice restored the alterations in the chromatin, along with the alterations in the DNA methylation and global histone acetylation, thereby suggesting the impact of the GM on the epigenetics-mediated transcriptional responses in the host. Additionally, adipose differentiation was enhanced upon propionate and butyrate administration to the stromal vascular fraction of porcine adipose tissue that suggest their potential inhibitory effect on the activity of the HDAC [209]. In another study, exposing the mice to SCFAs and other metabolites obtained from the *Akkermansia muciniphila* modulated the expression levels of the transcription factors, histone deacetylases, and other genes that were involved in satiety and lipid metabolism [210]. This association is further justified by the findings where obese subjects were reported to have reduced microbial diversity in their gut with the abundance of *Faecalibacterium prausnitzii* along with significantly low FFAR3 gene (FFAR3) methylation [211]. Further, low methylation in the TLR2 and TLR4 genes was associated with an increased weight and BMI along with a significant variation in the proportion of the lactic acid bacteria and Firmicutes: Bacteroidetes [211]. Further, deep sequencing analysis of DNA methylomes suggests a decisive association of bacterial predominance and epigenetic profiles [197]. Interestingly, the genes having variation in the methylation of the promotor region in the pregnant women with abundant microbes belonging to phyla *Bacillota* were demonstrated to be associated with the disorders such as inflammatory reactions, impaired lipid metabolism, obesity, etc., all of which are the risk factors for the development and progression of the CVDs [212]. An emerging set of evidence suggests that the fecal miRNAs has the potential to modulate the composition and organization of the GM, thereby suggesting the bidirectional association of the GM and epigenetics [212,213]. As discussed earlier, miRNA also find a very crucial place in pathogenesis of major CVDs, such as miR-133 in arrhythmias, and cardiac hypertrophy [94], miR-499 in cardioprotective effects and ischemia [96], miR-21, miR-23, miR-214, miR-126, miR-197, miR-208, and miR-223 in acute heart disease [97,98,99,100,101], miR-148 in lipid metabolism [102,103], and miR-425 in hypertension [105]. Although the studies are lacking to provide concrete evidence of how the interplay of the GM and epigenetics could modulate the CVDs, the accumulated evidence suggests that the composition of GM and epigenetics functioning are interrelated and have shown a direct association with obesity, body weight, BMI, and regulation of metabolism, which are the prime contributors to the development and progression of the CVDs.

## 5. Conclusions

**Figure 2:**
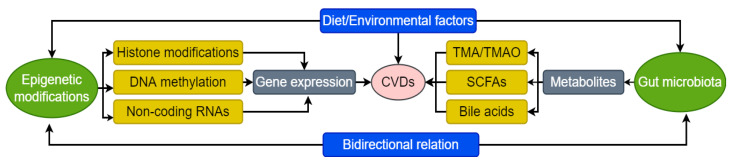
Depiction of the gut microbiota (GM)-epigenetics crosstalk in the pathogenesis of CVDs. DNA, deoxyribonucleic acid; RNAs, ribonucleic acids; CVDs, cardiovascular diseases; SCFAs, Short-chain fatty acids; TMA, trimethylamine; TMAO, trimethylamine-*N*-oxide.

CVDs are one of the leading causes of “sudden death” globally. Although complicated lifestyle, environmental exposure, and genetic factors are well-recognized etiological factors for the development of CVDs, the underlying pathological progression of these complications remains poorly understood. It is very difficult to predict the onset and development of CVDs well in time, which makes these disorders one of the leading causes of mortality and morbidity throughout the world. The overall conclusion of the review has been summarized in Figure 2.

Consistent high-end research work in recent years has unravelled the role of intestinal microbiota (the ecological hamlet of commensal, symbiotic, and pathogenic microbes) in several disorders, which led to the development of a ‘microbiome hypotheses’. Currently, it is well recognized that the vast communities of microorganisms that colonize the intestine play an important role in maintaining host homeostasis and influence complex pathologies in the state of dysbiosis. The host-microbiome association is a complex and challenging phenomenon and alternation in the composition of our gut microbiome and its metabolites contribute directly to the pathogenesis of CVDs. The GM generates several bioactive molecules including trimethylamine, trimethylamine-N-oxide, bile acids, fatty acids, etc., and influence cholesterol, coprostanol, peptidoglycan, immunity, and inflammatory pathways. The bioactive molecules and the pathways influenced by GM are predisposed by nutritional and lifestyle factors and may alter the cardiovascular dynamics and pathogenesis of CVDs. However, a complete understanding of GM, microbial genome, host genotype, diet, and CVDs interaction is a highly complicated process and is still a subject of research. 

Epigenetics and factors regulating epigenetic markers have also gained considerable attention in the recent past in explaining the pathogenesis of CVDs. Epigenetics was initially considered important for the pathogenesis of CVDs due to its direct influence on inflammatory reactions and vascular impairments. However, evidence accumulated in the recent past suggests that a significant number of modifications are involved in the development and progression of CVDs, which include histone modifications, DNA methylation, chromatin remodeling, microRNA, etc. Epigenetics and regulating factors play a vital role in the pathogenesis of complications such as diabetes, hypertension, cardiac hypertrophy, heart failure, myocardial infarction, and congestive heart failure, etc., besides being associated with the risk factors of CVDs such as smoking, aging, etc.

Both epigenetics and GM are governed by environmental, dietary, and genetic factors, suggesting a potential interaction between them. The existence of a bidirectional relation between the gut microbiota and epigenetics suggests that both of them can work in synchrony to modulate the disease representation and its pathogenesis. This is further established by the fact that the metabolites generated from the GM (such as SCFA etc.) are crucial not only for the normal physiological functioning of the body but also for the efficient working of the enzymes such as acetyltransferase, deacetylases, and methyltransferases that are involved in the epigenetic regulation of gene expression. Moreover, both of these are capable of providing us with numerous targets that can be exploited for the early diagnosis, prevention, and therapeutics of CVDs. Currently, there is no literature that implicates GM and epigenetics connection in CVD pathology. In-depth analysis of this connection is required and could result in the identification of novel therapeutic targets and help predict the onset of these disorders.

Another important thing to mention here is the need to discuss critical issues and the necessity to develop well-defined experiments before claiming that some microbes are or are not beneficial or even deleterious. As stated in the manuscript majority gut microbiota-based studies are correlative, meaning the researchers find a correlation/association of a specific microbe (i.e., bacteria) with a disease as potentially beneficial or deleterious. And the majority of the time, we jump to the conclusion and infer positive or negative effects without thorough investigations. The challenge lies in precisely pinning the role of the said microbe on the onset of the disease or conversely its beneficial impact.

These shortcomings partly come from the fact that it is difficult to culture some bacteria. Moving from identifying the presence of a microbe by sequencing to isolating it and then developing complex models for investing its effects is a herculean task. Although advancements in culturomics have made a significant leap, the identification of anaerobic bacteria remains time-consuming and troublesome. Another obstacle is mimicking the quantitative mixture of microbes for in vivo testing. On top of that, our knowledge of the role of any microbe in a complex community such as the gut microbiota is minimal.

## Data Availability

Not applicable.
